# Case series of keratitis in poultry abattoir workers induced by exposure to the ultraviolet disinfection lamp

**DOI:** 10.1186/s40557-015-0087-7

**Published:** 2016-01-15

**Authors:** Do-Hyeong Kwon, Jai-Dong Moon, Won-Ju Park, Won-Yang Kang, Soo-Hyeon Kim, Hyeong-Min Lim, Ji-Sung Ahn, Hong-Jae Chae

**Affiliations:** Department of Occupational and Environmental Medicine, Chonnam National University Hwasun Hospital, 322, Seoyang-ro, Hwasun-eup, Hwasun-gun, 519-763 Hwasun, Jeollanam-do Korea

**Keywords:** Photokeratitis, Poultry abattoir, Ultraviolet, South Korea

## Abstract

**Background:**

An outbreak of eye diseases occurred among workers at a poultry abattoir in South Korea from December 2012 to June 2013. An epidemiological investigation of the causative agent was conducted. The workers were given a special health examination and workplace environmental monitoring was performed. Workers with ocular symptoms subsequently underwent an ophthalmic examination.

**Case Presentaion:**

From a total of 41 workers, 26 (63.4 %) were diagnosed with keratoepitheliopathy by ophthalmic examination. Environmental monitoring of the workplace revealed that the ultraviolet (UV) apron-disinfection lamp had not been turning off at the set times, and so the workers’ faces had been exposed to UV radiation. Effective radiation dose measurement showed a UV-B exposure of 7-30 μW/cm^2^, and a UV-C exposure of 40-200 μW/cm^2^; both values exceed the occupational exposure limits. The outbreak ceased after the lamp was repaired.

**Conclusions:**

This case shows that inappropriate use of the UV disinfection lamp can cause mass photokeratitis. In order to prevent this, the UV disinfection lamp must be checked regularly, workers must be educated on the health effects of UV radiation, and appropriate eye protection must be worn.

## Background

Ophthalmologic diseases in industrial workplaces comprise as much as 5–20 % of all occupational diseases [1]. Among occupational ophthalmologic diseases of the cornea or conjunctiva, ocular trauma occupies the highest proportion—reported to account for 12.7–21.9 % of all industrial accidents in Korea, and 5–19 % of those in America [1, 2]. Most ocular trauma involves damage to the cornea or conjunctiva of the eyes. Other kinds of ocular trauma include post-trauma infection, corneal damage by ultraviolet (UV) radiation, burns, blunt ocular trauma, and eye perforation damage [3]. The causes of ocular trauma include direct damage by foreign substances, damage by chemical substances, cataract caused by hazardous light, and dry eye and pain caused by ergonomic problems such as computer monitor tasks [4, 5].

UV radiation is electromagnetic radiation with a wavelength of 100–400 nm. It is classified into UV-A (wavelength 400–320 nm), UV-B (wavelength 320–290 nm), and UV-C (wavelength 290–100 nm) depending on the range. UV radiation with a relatively short wavelength (<295 nm) is mostly absorbed by the anterior segment of eye; this causes damage to the cornea and conjunctiva, resulting in photokeratitis, particularly in welders [6, 7]. UV radiation with a relatively long wavelength, such as UV-A or UV-B, has an effect on the lens, causing cataract in cases of long-term exposure. Furthermore, pterygium is strongly correlated with this type of UV radiation [1].

This investigation was performed by the request of Ministry of Labor and employer. Workers at a poultry abattoir began experiencing mass ocular symptoms, including stinging eye, eye pain, and teardrops, from December 2012 to Jun 2013. We attempted to establish the cause through epidemiological investigation, and stipulated certain precautions to be taken in future to prevent it.

### Epidemiological investigation: case series

**Fig. 1:**
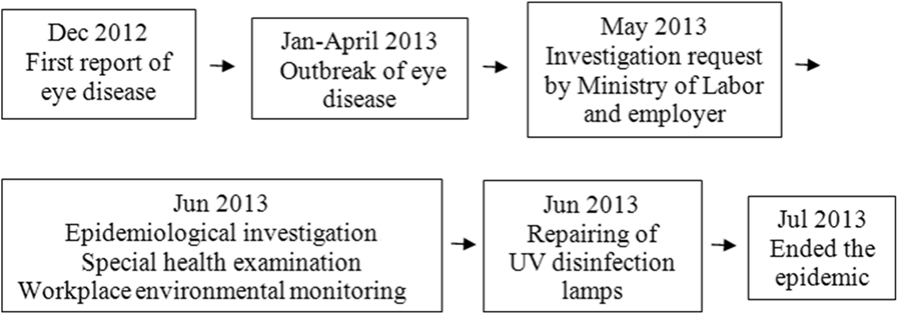
Timeline of epidemiological investigation

A mass outbreak of ocular symptoms occurred from December 2012 to June 2013 among the 41 production workers at a poultry abattoir located in Jeollanam-do Province in Korea. None of the office workers had any symptoms (Table 1). A special health examination was therefore performed on June 20, 2013. The examination included a complete blood cell count, immunoglobulin A (IgA) and immunoglobulin E (IgE) tests, plain chest radiography, and a pulmonary function test. Ophthalmologists conducted slit-lamp microscopy, as well as examinations to determine visual acuity, intraocular pressure, tear function, and ocular surface disease index (Fig. 1).

**Table 1:** General characteristics of the workers (*n* = 41)

Characteristics	*n* (%)
Sex	
Men	13 (31.7)
Women	28 (68.3)
Age (years)	
30-39	7 (17.1)
40-49	13 (31.7)
50-59	14 (34.1)
60-69	7 (17.1)
Work duration (years)	
0-4	19 (46.3)
5-9	13 (31.7)
10≥	9 (22.0)
Process	
Mooring	8 (19.5)
Cutting of meat	9 (21.9)
Evisceration and cleaning	10 (24.4)
Selection	14 (34.2)

Workplace environmental monitoring was performed for chemical substances and UV exposure in the workplace between June 20 and June 26, 2013. In order to identify which process (if any), and which task within said process, was the cause of the ocular symptoms, a chi-square test was performed. Specifically, this test was used to establish the symptom frequency and keratoepitheliopathy frequency of each work process, and these were then compared. In all analyses, p-values less than 0.05 were regarded as statistically significant. All statistical tests were performed using SPSS version 18.0 (SPSS Inc., Chicago, IL, USA).

## Case presentation

### The case

**Patient information:** Female, 51 years old

**Chief complaint:** Eye congestion, foreign body sensation

**Past history:** No remarkable past medical history

**Smoking history and alcohol history:** None

**Occupational history:** Meat cutting (duck parts). Worked since July 1, 2009.

**Present illness:** Stinging eyes and foreign body sensation had occurred from mid-December, 2012; eye congestion had also occurred frequently. Symptoms became worse in the presence of bright light. Ocular symptoms mostly occurred later in the working day and after work. Skin exfoliation and redness was observed around the eyes and on the face.

**Physical examination:** Mild hyperemic conjunctiva (+), erythematous diffuse patches (+), facial reddish papule (+), pruritic and stinging sense (+).

**Laboratory findings:** A peripheral blood examination revealed the following: hemoglobin level 11.9 g/dL, hematocrit 35.9 %, white blood cell count 4,500/mm^3^, and platelet count 180,000/mm^3^. According to an immunoassay, IgA was 214.0 mg/dL, IgE was 58.80 IU/ml. All these lab findings were within the normal range.

**Pulmonary function test findings:** A pulmonary function test revealed the following: functional vital capacity (FVC) was 3.68 L (115.4 %), forced expiratory volume in one second (FEV_1_) was 3.00 L (124.5 %), the ratio of FEV_1_ to FVC was 81.52 % (108.8 %). All findings were within the normal range.

**Medical imaging findings:** There were no significant findings upon plain chest radiography.

**Fig. 2:**
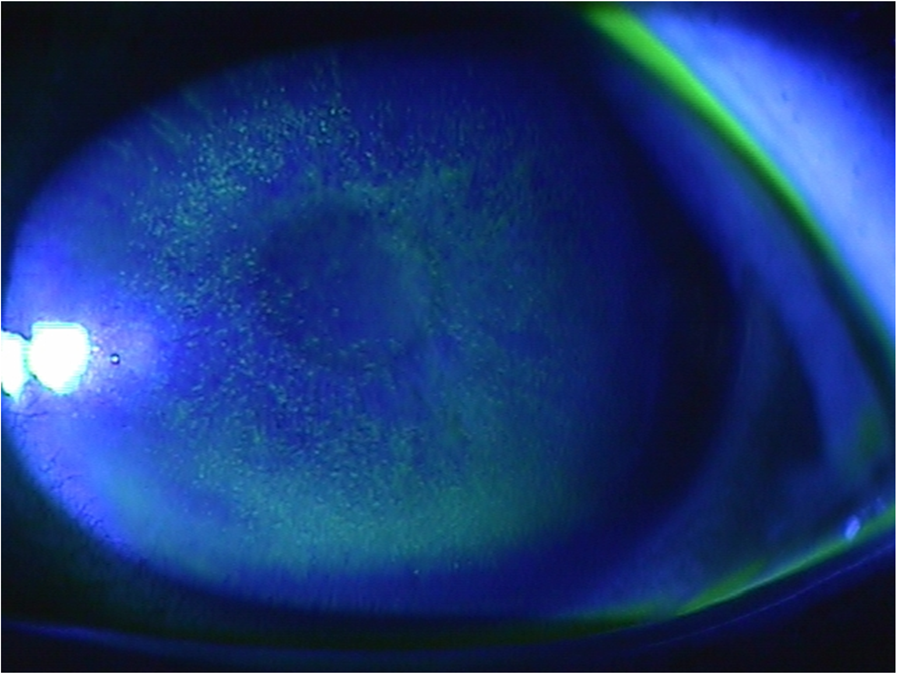
Example of keratoepitheliopathy observed by fluorescein staining

**Fig. 3:**
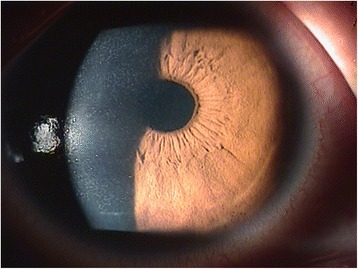
Example of keratoepitheliopathy observed by slit-lamp microscopy

**Ophthalmology findings:** Visual acuity was 0.1 in the right eye and 0.08 in the left. Maintained anterior chamber or anterior chamber cells were not observed in either eye. Intra ocular pressure was 14 mmHg in the right eye and 14 mmHg in the left. Schirmer`s test result was 20 mm in the right eye and 18 mm in the left. Diffuse corneal damage was observed (Figs. 2 and 3). On the basis of these results, keratoepitheliopathy was diagnosed.

### Special health examination

At the time of medical examination by interview, 26 out of a total of 41 workers (64 %) were complaining of ocular symptoms (stinging eye, teardrops, *etc*.). Moreover, skin symptoms (skin exfoliation, redness) were found in 4 workers (9 %) upon physical examination. Ocular symptoms mostly occurred immediately after finishing work or after going home, and skin symptoms around the eyes often occurred in workers. None of the workers had an abnormal complete blood cell count, and all Ig A and Ig E tests were normal. In addition, plain chest radiography and pulmonary function tests showed no abnormal findings in any of the workers. An ophthalmic examination yielded normal conjunctival findings in all workers; however, 26 out of 41 workers (63.4 %) were diagnosed with keratoepitheliopathy (Table 2). That is, all of the workers who had complained of the symptoms were diagnosed with keratoepitheliopathy.

**Table 2:** Symptoms and test results of the workers (*n* = 41)

Symptoms and test results	*n* (%)
Symptoms	
Ocular symptoms	26 (63.4)
Skin symptoms	4 (9.7)
Respiratory symptoms	0 (0.0)
Keratoepitheliopathy diagnosis	26 (63.4)

## Exposure assessment

### Symptoms classification by process

The abattoir processed chickens and ducks, and process flow involved 4 steps in the following order: mooring, cutting of meat, evisceration and cleaning, and selection.

There were no significant differences among the processes with regard to the frequencies of workers who complained of ocular symptoms (*p* = 0.142) or skin symptoms (*p* = 0.112) (Table 3). These results confirmed that the symptoms were not caused by any specific process.

**Table 3:** Symptoms by work process

Variables	n	Skin symptoms *n* (%)	*p*-value*	Ocular symptoms *n* (%)	*p*-value*
Mooring	8	2 (25.0)	0.112	3 (37.5)	0.142
Cut of meat	9	5 (55.5)		8 (88.8)	
Evisceration and cleaning	10	1 (10.0)		5 (50.0)	
Selection	14	3 (21.4)		10 (71.0)	
Total	41	11(26.8)		26(63.4)	

*p-value was calculated using the chi-square test

### Keratoepitheliopathy by process

There were no significant differences among the processes in terms of keratoepitheliopathy patient frequency (*p* = 0.393) (Table 4).

**Table 4:** Keratoepitheliopathy by work process

Variables	N	Keratoepitheliopathy n (%)	*p*-value*
Mooring	8	4 (50.0)	0.393
Cut of meat	9	7 (77.7)	
Evisceration and cleaning	10	6 (60.0)	
Selection	14	9 (64.2)	
Total	41	26(63.4)	

^*^ *p*-value was calculated using the chi- square test

### Workplace environmental monitoring

Exposure to chemical substances and UV radiation was measured. Workplace environmental monitoring was conducted, focusing on 3 processes: cleaning, cleaning finish, and evisceration and hooking, wherein chlorine, hydrochloric acid, and sodium hydroxide was measured. The workers were not exposed to all 3 chemical substances in all processes, and even in processes where they were exposed, the exposure was well below the permitted limit (Table 5).

**Table 5:** Workplace environmental monitoring of chemicals by process

Variables	Volume (L)	Measuring position	Measuring time	Measurements (ppm)	Exposure limit	Measurement Way
Cleaning						
Chlorine	320.4	RS	10:01 ~ 15:21	0.0108	0.5 ppm	Filtration/IC
Hydrochloric acid	50.8	RS	11:15 ~ 15:17	ND	1.0 ppm	Solid/IC
Sodium hydroxide	30.0	RS	11:17 ~ 11:32	ND	2.0 mg/m^3^	Filtration/AA
Cleaning finish						
Chlorine	324	RS	10:01 ~ 15:21	0.0107	0.5 ppm	Filtration/IC
Hydrochloric acid	55.9	RS	11:15 ~ 15:17	ND	1.0 ppm	Solid/IC
Sodium hydroxide	30.0	RS	11:17 ~ 11:32	ND	2.0 mg/m^3^	Filtration/AA
Evisceration						
Hydrochloric acid	52.0	RS	11:15 ~ 15:17	ND	1.0 ppm	Filtration/IC
Sodium hydroxide	30.0	RS	11:17 ~ 11:32	ND	2.0 mg/m^3^	Filtration/AA

*RS* Regional samples, *ND* Not detected, *IC* Ion chromatography, *AA* Atomic absorption

Workers had none of respiratory symptoms or mucous membrane irritation symptoms that can manifest after exposure to the above chemical substances. In addition, there were no abnormal findings in the pulmonary function test, so that it was not possible to explain the skin and ocular symptoms in terms of exposure to the above chemical substances. What is more, while chemical substances were only used in the department of cleaning and evisceration, keratoepitheliopathy appeared in all departments. Therefore, it was clear on the basis of these findings that exposure to chemical substances was not the cause of the outbreak.

Photokeratitis caused by UV radiation was also suspected – based on the ocular and skin symptoms of the workers. For this reason, the effective radiation dose from the UV disinfection lamp was measured. The apron disinfection lamp at the 1^st^ floor was measured and it was found that it emitted UV-B at 15-30 μW/cm^2^ and UV-C at 40-160 μW/cm^2^ with a 5–10 cm measuring distance. The lamp at the 2^nd^ floor emitted UV-B at 7-10 μW/cm^2^ and UV-C at 40-200 μW/cm^2^. On both floors, these measurements exceeded the exposure limit (American Conference of Governmental Industrial Hygienists [ACGIH] threshold limit values: 10 min at 5 μW/cm^2^, 5 min at 10 μW/cm^2^; Table 6) [7].

**Table 6:** Workplace environmental monitoring of ultraviolet C by equipment

Variables	UVC effective radiation (μW/cm^2^)
First floor	
Screening package	3.0 ~ 3.5
Disinfection lamp	40.0 ~ 160.0
Second floor	
Screening package	3.0 ~ 4.5
Disinfection lamp	40.0 ~ 200.0

UVC: Ultraviolet C, Screening package: Chicken package machine by size and emit ultraviolet in the workplace

**Fig. 4:**
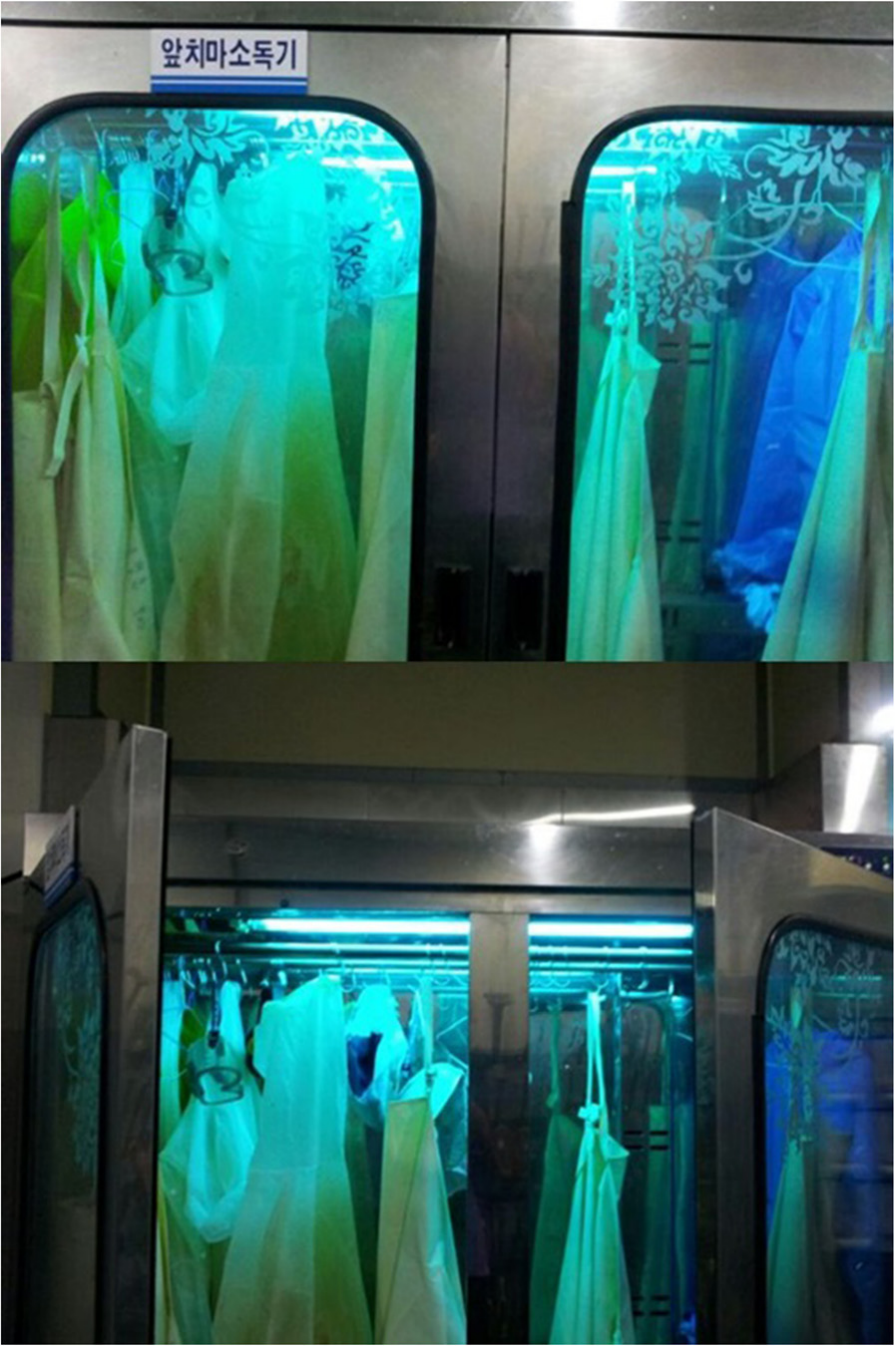
Dysfunctional ultraviolet disinfection lamp: Since the lamps did not turn off automatically, even when the doors were opened, the faces of workers were exposed to ultraviolet radiation

The apron disinfection lamps and UV lamps, in the factory are set to turn off automatically when the door of the disinfection equipment is opened. However, this setting was found to be malfunctioning, and the UV lamps of the disinfection equipment were remaining on when the door was opened. Since workers in all processes need to wear and remove aprons several times a day, it was determined that each individual may have been exposed to the UV radiation of the apron disinfection lamps from a short distance for at least 10 min a day (Fig. 4). It was therefore possible that all workers had been exposed to UV radiation for a significant amount of time, thus potentially explaining the ocular symptoms and keratoepitheliopathy that had occurred in a broad range of workers regardless of process. Furthermore, this was the likely explanation for the sunburn-like skin lesions on the face by exposure to UV. Further evidence was provided when workers’ ocular and skin symptoms improved after removal of the UV disinfection lamps. Taken together, it was highly potential that the mass photokeratitis that had occurred in the poultry abattoir was caused by exposure to UV radiation from the apron disinfection lamps.

## Discussion

UV radiation is divided into 3 subtypes: UV-A (400–320 nm), UV-B (320–290 nm), and UV-C (290–100 nm). Continuous exposure to UV radiation can have an effect on the skin. UV-A reaches into the deep skin layer, and affects connective tissue and blood vessels; this may cause loss of elasticity and premature ageing. UV-B stimulates formation of new melanin, inducing dark pigments, and a high dose of UV-B causes sunburn and increases the chance of skin cancer. UV-C can cause both erythema and severe burns in the epidermis, and can lead to skin cancer in a similar manner to UV-B [6].

UV radiation can cause photoconjunctivitis and photokeratitis in the eyes. These symptoms are often accompanied by pain and a sunburn-like pattern on sensitive skin, although they have not been known in themselves to be related to long-term damage. Having said that, continuous exposure to UV radiation may cause pterygium, cataract, melanoma, and even basal cell carcinoma [7].

UV disinfection lamps are mostly used for the destruction of micro-organisms. They have germicidal effects and emit UV radiation with a wavelength 260 nm, which is the optimum value for absorption by the DNA of micro-organisms. Therefore, when these lamps are concerned, it is mainly UV-C in the corresponding wave area that can affect the human body [8]. Although UV radiation has weak penetration, it has strong energy due to its short wavelength. It therefore promotes chemical reactions, oxidates organics, and killes micro-organisms by destroying DNA in the nuclei and mitochondria, thus preventing the respiration and multiplication of micro-organisms [7].

Photokeratitis or UV keratitis can occur with continued natural exposure (*e.g*. intense sunlight) or artificial exposure (*e.g.* electric arc during welding) to UV radiation. Symptoms include an increase in teardrop production, foreign body sensation, ocular pain, contraction of pupils, and eyelid convulsion, and the condition is definitively diagnosed when spot shapes appear under UV irradiation of fluorescein staining of the eye [9]. Exposure can occur in welding when the operator neglects to wear protective equipment, or when UV radiation is reflected by ice or snow. Both instances may lead to keratitis. Although anesthetic eye drops can be applied as a treatment, long-term use of such medications can result in corneal ulcer or blindness, so is not recommended. In contrast, although NSAID-type eye drops can be applied to control pain, their efficacy has not yet been confirmed. When pain is severe, oral analgesics can be administered. When the cause of keratitis is removed, the condition is known to resolve within 24–72 h [10].

Photokeratitis caused by UV radiation has been reported in multiple studies outside Korea. For example, Kirschke et al., reported that exposure to a metal halide lamp used in a gymnasium caused photokeratitis and UV-radiation burn, and Verma et al., reported that exposure to UV radiation caused by incorrect control of an UV disinfection lamp in an aquarium caused photokeratitis [8, 11–13]. In addition, Banerjee reported that mass photokeratitis occurred in 150 individuals after exposure to unprotected UV light at a cattle stock market in the United Kingdom [14].

The present study was implemented in response to a request for an epidemiological investigation into ocular and skin symptoms that were occurring in workers at a poultry abattoir over a 6-months period. We performed a complete blood cell count, an immunoassay, plain chest radiography, a pulmonary function test, an ophthalmologic consultation, and an ophthalmic test. All workers studied showed normal findings in the complete blood cell count, immunoassay, plain chest radiography, and pulmonary function test, by which allergic disease or infectious disease were excluded. Workplce environmental monitoring was performed on chlorine, hydrochloric acid, and sodium hydroxide, as there was a possibility of exposure to chemical substances. However*,* across all processes, exposure to these substances was either null or below the exposure limit.

Since there was no respiratory or mucous membrane irritation, and also no differences among the processes or departments in terms of frequency of ocular and skin symptoms, the possibility of exposure to chemical substances could be excluded. On the other hand, when the working environment was monitored for the use of UV disinfection lamps, it was found that exposure limit had been exceeded. Since workers across all processes were using the UV disinfection lamps, and showed ocular and skin symptoms that are consistent with exposure to UV radiation, it could be concluded that the keratitis outbreak had been caused by exposure to UV radiation from the malfunctioning UV disinfection lamps. No ocular or skin symptoms occurred after repair of the UV disinfection lamps, and so the outbreak ceased.

In order to prevent mass photokeratitis caused by UV disinfection lamps, as in the present case, both employers and workers must make an effort. Employers need to place caution marks and labeling on UV disinfection lamps to ensure workers recognize the risk. Moreover, UV disinfection lamps need to be checked regularly, and it must be confirmed at installation whether the disinfection lamps have been installed properly. It is also necessary to use lamps that fulfill the international standards and which turn off automatically, and when damage to the lamps is identified during a check-up, the power must be turned off immediately, and workers need to be separated from the lamps. What is more, the working environment must be monitored regularly for UV disinfection lamps. Lastly, workers must be required to maintain a proper distance from the UV disinfection lamps while working to avoid long-term exposure, and must not ignore ophthalmologic symptoms, but must visit the ophthalmology clinic immediately. They must be familiar with the effects of UV radiation on their health, and be aware of the corresponding risks.

## Conclusions

Currently, UV disinfection lamps are widely used not only in poultry abattoirs, but also in restaurants and various other business places. Hence, keratitis caused by UV radiation, as in the present case, can happen anywhere. Since mass photokeratitis caused by UV radiation is a preventable disease, a regular check-up should be performed in the workplace based on an understanding of UV disinfection lamps. In addition, to prevent photokeratitis, workers must wear eye protection devices and receive education on the health effects of UV radiation.

## Consent

Written informed consent was obtained from the patient for publication of this case report and any accompanying images. A copy of the written consent is available for review by the Editor-in-chief of this journal.
